# Scarcity Value Assessment of Ecosystem Services Based on Changes in Supply and Demand: A Case Study of the Yangtze River Delta City Cluster, China

**DOI:** 10.3390/ijerph191911999

**Published:** 2022-09-22

**Authors:** Xiaoping Zhou, Lan Yang, Xiaokun Gu, Lufa Zhang, Li Li

**Affiliations:** 1 School of Government, Beijing Normal University, Beijing 100875, China; 2 School of International and Public Affairs, Shanghai Jiaotong University, Shanghai 200030, China; 3 China Institute for Urban Governance, Shanghai Jiaotong University, Shanghai 200030, China; 4 Institute of Healthy Yangtze River Delta, Shanghai Jiaotong University, Shanghai 200030, China

**Keywords:** ecosystem services, scarcity value, land-use change, Yangtze River Delta urban agglomeration, spatio-temporal characteristics

## Abstract

Rapid urbanization and economic development have resulted in a mismatch between the supply and demand of ecosystem services. The theoretical value of ecosystem services (ESTV) is not suitable for determining ecosystem service compensation, posing challenges for integrated regional ecological development. A scarcity value model was used to analyze the influence of changes in supply and demand on the scarcity value of ecosystem services (ESSV) in the context of land-use change. The spatio-temporal distribution characteristics and trends of the ESSV from 2010 to 2020 were assessed in the Yangtze River Delta (YRD) urban agglomeration in China, and the driving factors were analyzed to provide theoretical guidance for horizontal ecological compensation across regions. The results show the following: (1) In the scenario that did not consider the impact of supply and demand changes on the scarcity value, the total ESTV decreased by 8.67% from 2010 to 2020, and high-value areas shifted to the west and south, whereas low-value areas shifted to the central and northern region and the Jiangsu, Zhejiang, and Shanghai Ringbelt. The ESTV was low in Shanghai and Jiangsu and high in Zhejiang. (2) In the scenario that considered changes in the supply and demand of ecosystem services, the ESSV increased from RMB 213 million in 2010 to RMB 1.323 billion in 2020; an increase of 521.13%. The scarcity value showed high variability within the provinces, with a larger difference between Zhejiang and Jiangsu and a smaller difference between Anhui and Shanghai. The ESSV was higher in counties with increased urbanization and high population density and lower in counties with slower economic growth and fewer people. (3) Regional ecological integration planning and management should be strengthened, and the ESSV might be considered as the reference standard for ecological compensation. The ESSV showed that spatio-temporal heterogeneity might guide the conversion from ecological resources to ecological capital and promote the regulatory role of market mechanisms to achieve horizontal payments for ecosystem services across regions.

## 1. Introduction

Urbanization is inevitable given the current economic and social development. Six major urban agglomerations exist worldwide. Half of the world’s population is living in cities (prediction for 2020), and the proportion will rise to 67% in the next 30 years [1]. Increased urbanization and regional economic growth have destroyed 60% of global ecosystems during the last 50 years [2,3,4], and the loss of ecosystem services (ESs) is the most significant in urban agglomerations [5,6,7]. More people are facing the direct consequences of global climate change, such as an increase in the frequency of extreme weather events, the loss of terrestrial ecosystems and biodiversity [8], and the deterioration of urban environments [9,10], posing a threat to the health of the population. The Yangtze River Delta (YRD) urban agglomeration in China is growing rapidly, with an urbanization rate of 13% for the resident population, a 15.06% increase in total construction land, and a 36.09% decrease in the area of natural ecosystems in 2020 compared to 2010. Although the average forest cover is 33.23% [11,12,13,14], it is still lower than that of international metropolitan clusters [15]. A decline in the area of natural ecosystems and a rising population have created significant supply and demand conflicts for ESs, posing challenges to sustainable urban development and improved resident well-being.

ESs are defined as “the contributions of ecosystem structure and function (in combination with other inputs) to human well-being” [16]. They include supporting services, provisioning services, regulating services, and cultural services [2], which are crucial for sustainable urban development, cultural vitality enhancement, and healthy biosphere conservation. Action initiatives have been proposed at global, regional, and national scales (e.g., Human Sustainable Development Goals (SDGs), Millennium Ecosystem Services Assessment (MA), Building Ecological Civilizations, International Panel on Biodiversity and Ecosystem Services) to emphasize the critical role of ESs in improving human well-being, conserving biodiversity values, and supporting natural capital. Most countries and regions have government initiatives focused on ecological construction, establishing ecosystem management systems, and incorporating ecological protection into government performance assessment to meet the growing demand for ESs [17]; however, ecological construction is often hindered by investment constraints [18]. Furthermore, a region may pay the opportunity cost of development due to ecological construction and fall into poverty due to large investments, exacerbating social inequity and instability [19]. Ecological compensation (EC) is an effective strategy to protect ecosystems and balance ecological and socio-economic development and has been incorporated into the laws or policies of 56 countries [20]. Early EC models in China used top-down financial transfers and central government subsidies, primarily by the central and local governments [21], because they relied on policy support, frequently resulting in inefficient and lagging compensation when responding to diverse compensation targets. In 2013, the central government established the horizontal compensation system between regions and advocated for the integration of a market mechanism into EC. This model has increased the public’s ecological awareness, and the compensation concept has focused on the intrinsic ecological demand and supply potential of relevant stakeholders to ensure diverse funding measures and synergistic regional development [22]. However, the scope of the relevant stakeholders and the amount of EC remain unclear. Urbanization, population growth, and economic development have increased the scarcity of ESs by increasing the number of beneficiaries with a much greater willingness to pay for ecological services [23,24]. Scarcity in economics refers to the infinite desire of human beings versus the limited resources to satisfy human needs [25]. The scarcity of ESs, caused by changes in supply and demand, produces fluctuations in the value of ESs during urbanization [26,27]. When the scarcity value is considered, the total value of ESs may increase [28,29]. The scarcity value reflects the match between the supply and demand for ESs, provides theoretical guidance for inter-regional horizontal ecological compensation price (ECP)-setting, and compensates for the failure of theoretical values to guide market transactions.

Recent studies have primarily assessed the theoretical value of ecosystem services (ESTV) [30,31,32,33], using the equivalent value approach [34,35], integration analysis, and value transfer methods [36,37], without considering the effect of supply and demand fluctuations on the scarcity value of ecosystem services (ESSV). Studies have explored the importance of the scarcity of ESs, and a stochastic control theoretical framework has been developed to obtain a numerical value closely related to the scarcity value of the ES provided by lakes [25]. Barbier discussed the economic significance of ESs scarcity and advocated improving the economic and scientific analyses of ecological scarcity to evaluate the loss in benefits [38]. Sandhu et al. evaluated the impact of pollinator scarcity on agricultural production. The estimated economic loss to New Zealand’s agriculture was NZD $295–728 million annually due to a decline in pollinators [26]. Recent studies have constructed assessment models to assess the scarcity value of private and public ESs in Chinese cities and suggested that the major driver of the dramatic increase in ESSV was the sharp rise in the demand for public goods services [6,39]. The spatio-temporal interaction between urbanization and the change in ESSV in China was investigated to reveal the driving mechanism of ESSV [40,41]. Although the significance of the scarcity value of ESs and its driving mechanism are widely recognized, and the method has progressed from qualitative to quantitative evaluation, there is a knowledge gap between the scarcity value, ecological compensation, and conservation policies. Thus, it is critical to apply the theory of the scarcity of ESs to the ecological governance of countries. Due to the small proportion of urban ecosystems, most research has focused on natural landscape areas [32,42,43] and ecological watersheds [44,45]. However, ESs are in short supply and high demand by residents in urban areas, leading to a high scarcity of ESs. Therefore, there is an urgent need to assess the ESSV in urban agglomerations to provide theoretical guidance for urban green infrastructure construction and determine the cross-regional ECP.

The purpose of this study is to establish a method for assessing the ESSV in urban agglomerations based on changes in supply and demand and provide theoretical guidelines for the inter-regional determination of the ECP. First, we identify the ESs flowing across areas and establish an ESSV model based on changes in supply and demand. Second, we conduct a case study of the YRD urban agglomeration to examine the theoretical supply value and ESSV at the county scale to obtain the spatial pattern of the ESSV. Finally, based on the ESSV’s spatial characteristics and driving forces, we propose policy recommendations for ecological management and regional synergistic development in urban regions. The primary contributions of this paper include the following. (1) The proposed method of assessing the influence of changes in supply and demand on the value of ESs is an improved approach for valuing ESs to identify the scarcity value. (2) The spatial characteristics and driving factors of the ESSV in metropolitan clusters provide a theoretical basis for inter-regional horizontal ECP-setting and decision support for policymakers and land managers to achieve the cross-regional flow of ESs.

## 2. Theory and Data Sources

### 2.1. Theoretical Framework and Technical Route

Based on the classification of ESs by the MA, researchers have further classified ESs into in situ services, omni-directional services, and directional services according to the possible spatial relationships between service production areas (SPA) and service benefit areas (SBA). In in situ services, the ESs are provided and the benefits are obtained in the same location (e.g., soil formation, provision of raw materials). In omni-directional services, the ESs are provided in one location but benefit the surrounding landscape without a directional bias (e.g., pollination, carbon sequestration). In directional services, the ESs provide benefits at a specific location due to the flow direction (e.g., water regulation services and storm and flood protection) [46]. ESs provided in situ only benefit this region and do not affect or cannot be compensated for across borders. In addition, food production services can be traded in existing markets and do not need to be evaluated. Therefore, omni-directional and directional ESs that can flow across regions were selected for determining the regional horizontal EC (Table 1).

**Figure 1:**
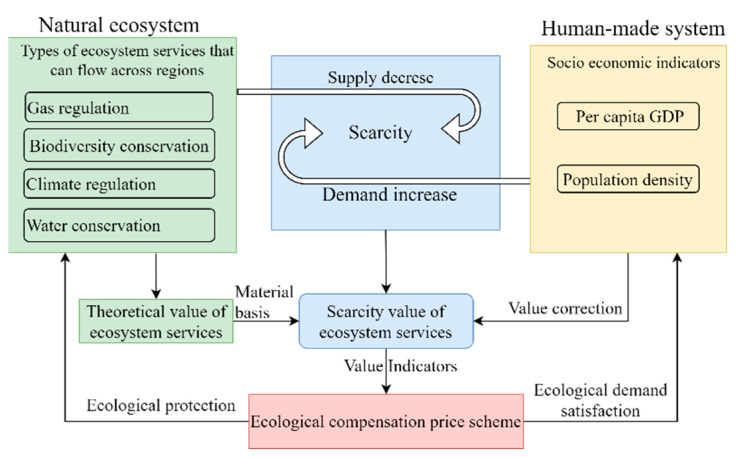
Theoretical analysis framework.

Scarcity is generated by a decline in ESs provided by natural ecosystems and an increase in the demand for ESs in anthropogenic systems; therefore, the ESSV is impacted by supply and demand. The theoretical value of ESs that flow across areas serves as the material basis and is adjusted by socioeconomic factors to obtain the scarcity value of ESs [47]. The per capita GDP and population density [6,39] are commonly used to revise the theoretical scarcity values. Therefore, the spatial distribution characteristics of the regional ESSV provide a scientific indicator for horizontal inter-regional ECP-setting. Regional horizontal ecological compensation can improve ecological sustainability and decrease the risk of ecosystem deterioration while addressing inhabitants’ increasing ecological requirements. The theoretical analysis framework of the ESSV to guide the ECP-setting is shown in Figure 1.

**Figure 2:**
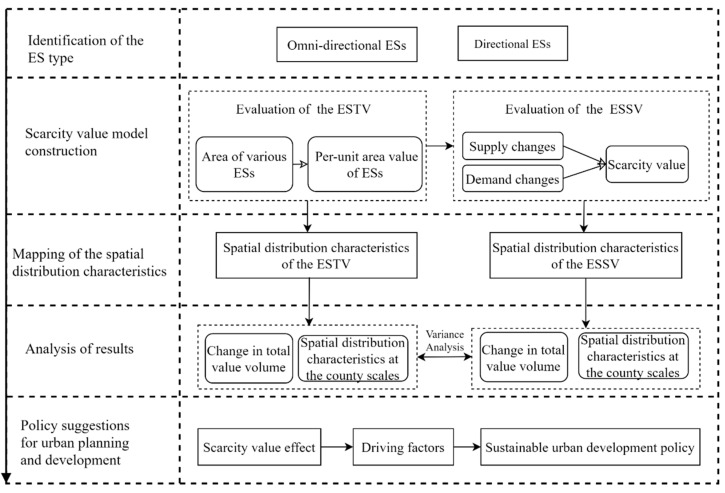
Technical Route.

The technical route is as follows (Figure 2). (1) Selecting the types of ESs that can flow across regions. (2) Constructing an ESSV model based on changes in supply and demand. The ESTV is evaluated and socio-economic indicators are chosen to adjust it to derive the ESSV. (3) Mapping the spatial distribution characteristics of the ESTV and ESSV. (4) The trend in the total value of the ESTV and ESSV and the spatial distribution characteristics are analyzed from 2010 to 2020 and the driving factors are determined. Policy recommendations are provided to develop urban green infrastructure and ensure integrated regional ecological development in the YRD city cluster.

### 2.2. Study Area

**Figure 3:**
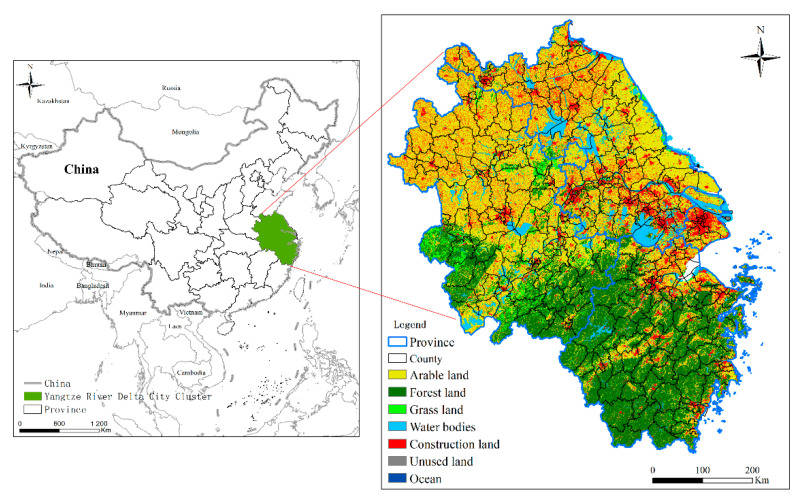
The geographical location of the Yangtze River Delta region and its land-use categories in 2020.

The YRD city cluster is located in the lower reaches of the Yangtze River in China (N31°, E121°), bordering the Yellow Sea and the East China Sea. It is located on an alluvial plain formed before the Yangtze River enters the sea, with many coastal ports along the river (Figure 3). Most of the YRD Plain is located 10 m above sea level, with a few small hills with elevations of 200–300 m. The average annual temperature is 13–18 °C, and the annual precipitation is 800–1600 mm. The area is influenced by the East Asian monsoon and has a pleasant climate. Most of the precipitation falls in summer. The YRD city cluster is an important region for China to participate in international competition, a leader in the Yangtze River Economic Belt, and one of the best-urbanized areas in China. According to the “Yangtze River Delta Regional Integrated Development Plan Outline” (http://www.gov.cn/( accessed on 20 March 2022)) approved by the State Council in 2019, the city cluster includes 41 prefecture-level cities and 305 counties in Shanghai, Jiangsu, Zhejiang, and Anhui provinces, covering an area of 358,000, accounting for 3.7% of the country’s area. In 2020, the resident population of the YRD region accounted for 12.1% of China’s population, the total regional GDP accounted for 24.1% of the national GDP, and the average urbanization rate was 73.26% [48]. Fifteen cities have a resident population of more than 5 million, and most of them (urban areas) have a resident population exceeding the criteria for large cities, with an overall large urban scale.

The YRD area has a high level of urbanization, yet the population density and economic development level vary by region. The conflict between ES deterioration and resident demand is intense, and the need for ecological development is pressing. Therefore, determining the scarcity of ESs is the theoretical basis for horizontal EC and decision-making for the integrated ecological and green development of the YRD region. In addition, the spatially differentiated characteristics of the ESSV portray the current situation of ESs in typical metropolitan areas, providing a case for assessing the ESSV in other metropolitan areas in China and globally.

### 2.3. Data Source

The socio-economic statistics used in this paper were obtained from the Shanghai, Zhejiang, Anhui, and Jiangsu Province Statistical Yearbooks [49,50,51,52]. The vector land-use data for the YRD City Cluster in 2010 and 2020 were provided by the Chinese Academy of Sciences Resource Science and Environmental Science Data Center [53]. The data were reclassified into 7 primary categories (arable land, woodland, grassland, water bodies, construction land, unused land, and ocean) and 27 secondary categories according to the national land-use classification system.

## 3. Methodology

### 3.1. Assessing the Theoretical Value of Ecosystem Services

First, the ES value coefficient was adjusted based on actual conditions in the YRD using the unit value transfer method [54,55] and expert consultation [47]. Then, the value of ESs per unit area was calculated; it was defined as 1/7 of the total economic value of food production services from cultivated land [56]. We assigned the per-unit yuan values to the ESs provided by the land-use categories in the YRD by multiplying the locally adjusted coefficients by the reference value of the food production from the cultivated land per hectare.

#### 3.1.1. The Ecosystem Service Value Coefficients

We adjusted the ES value coefficients to reflect local conditions from two aspects. First, the biomass factor was revised. In general, the greater the biomass, the greater the ESs are [57]. We adjusted the value coefficients for arable land, woodland, grassland, water bodies, desert, and construction land using the ratio of the regional to national average grain yield and the ratio of the YRD’s average net primary productivity (NPP) of the forest ecosystem to the corresponding data at the national scale. Second, the value coefficients for the secondary land-use classes were assigned. Since Xie et al. used relatively coarse land-use categories, the practical application was limited. For example, the arable land categories were not divided into paddy fields, irrigated land, dry land, and garden land, and the ES values of these types were relatively different. Some scholars have subdivided the ES values of land-use categories based on expert consultation and existing studies [58]. We assigned the value coefficients to the secondary classes of land-use categories following Tang (2010).

#### 3.1.2. The Per-Unit Value of Ecosystem Services

The value of the ESs equivalent factor per unit area is the economic value of the natural food production of one hectare of farmland in that year. The per-unit value of ESs was set as 1/7 of the total economic value of food production services from arable land [45], as defined in Equation (1).

(1) $$E_{a}=\frac{1}{7}\overset{n}{\underset{i=1}{\sum }}\frac{m_{i}p_{i}q_{i}}{M}\;\;\left(i=1,\;2,\;3,\ldots ,\;n\right)\;$$

where $E_{a}$ is the per-unit value of ESs in the YRD (yuan/${\mathrm{hm}}^{2}$); i is the major food crop; $p_{i}$ is the national average price of the th food crop (yuan/kg); $q_{i}$ is the yield per unit area of the *i*th food crop (kg/${\mathrm{hm}}^{2}$); is the area of the th food crop nationwide (${\mathrm{hm}}^{2}$); M is the total area of all food crops nationwide (${\mathrm{hm}}^{2}$); and 1/7 is the economic value provided by a naturally functioning ecosystem without human cost inputs, which is 1/7 of the value of food production services provided by a unit area of farmland.

#### 3.1.3. The Theoretical Value of Ecosystem Services

The ESTV is an estimate of the physical supply of ESs and does not consider the scarcity affected by changes in supply and demand. Based on Costanza’s theory, the physical supply of ESs can be calculated with Equation (2):

(2) $$ESTV_{s,c,t}=A_{c,t}\times VC_{s,c}$$

where $ESTV_{s,c,t}$ is the value of the ES category *s* of land use *c* at time *t*, *A_c,t_* is the area of land use *c* at time *t*, $VC_{s,c}$ is the per-unit value coefficient of ESs in land use c (Table 2 and Table 3), and t ∈{2010, 2020}.

### 3.2. Assessing the Scarcity Value of Ecosystem Services

#### 3.2.1. Scarcity Factor Adjustment for Ecosystem Services

Scarcity in economics is relative to the infinity of human desire [22,59]. The satisfaction of human desire can only be achieved by consuming a product, but there are limitations due to limited resources, namely scarcity. According to the theory of marginal utility, the value of goods depends on utility and scarcity; marginal utility is the added satisfaction derived by a consumer by consuming an additional unit of a good or service [60]. A decrease in the supply of ESs due to rapid urbanization is driven by land-use change [61,62]. Conversely, an increase in the demand for ESs is affected by increases in the population and wealth and changes in spending preferences [24,63]. The YRD region has a high level of urbanization and economic development; thus, the ESSV should be significantly higher than the national average. The ESSV in the YRD region increased during the study period, leading to an increase in the unit value of ESs. Therefore, it is necessary to adjust the scarcity factor for ES valuation.

We defined the scarcity of ESs as the difference between the limited ESs and the theoretically limitless demand by humans. The ESSV represents an increase in the marginal utility an individual obtains from consuming an additional unit of ESs due to the increasing scarcity of ESs. Following Bryan et al. (2018), we assumed that the per-unit ESSV represented the price and used Equations (3)–(7) to calculate the ESSV coefficient in the YRD.

(3) $$\Delta p_{s,t1}^{Sup}=\Delta Q_{s,t1}^{Sup}\times \Delta P_{s,t1}^{Sup}$$

(4) $$\Delta Q_{s,t1}^{Sup}=-\frac{ESTV_{s,c,t1}-ESTV_{s,c,t0}}{ESTV_{s,c,t0}}$$

where $\Delta p_{s,t1}^{Sup}$ is the coefficient of the supply-driven relative change in the scarcity value, $\Delta Q_{s,t1}^{Sup}$ is the proportional change in the quantity supplied, $\Delta P_{s,t1}^{Sup}$ is the relative change in the scarcity value resulting from a decrease in the supply of ESs, and t1 ∈{2010,2020}.

(5) $$\Delta p_{s,t1}^{Dem}=\Delta Q_{s,t1}^{Dem}\times \Delta P_{s,t1}^{Dem}$$

(6) $$\Delta Q_{s,t1}^{Dem}=\left(WTP_{s,t1}-WTP_{s,t0}\right)/WTP_{s,t0}$$

(7) $$WTP_{s,t}=POP_{t}\times GDP_{t}\times \varepsilon_{s,t}$$

where $\Delta p_{s,t1}^{Dem}$ is the coefficient of the demand-driven relative change in the scarcity value, $\Delta Q_{s,t1}^{Dem}$ is the proportional change in the quantity demanded, $\Delta P_{s,t1}^{Dem}$ is the relative change in the scarcity value resulting from an increase in the demand for the ESs, $WTP_{s,t}$ is the willingness to pay for ESs at time *t*, *POP_t_* is the total population (persons), $GDP_{t}$ is the real per capita GDP at time t, and $\varepsilon_{s,t}$ is the income elasticity of demand for each ES at time t.

#### 3.2.2. Price Elasticity of Ecosystem Services with a Change in the Scarcity Value

**Figure 4:**
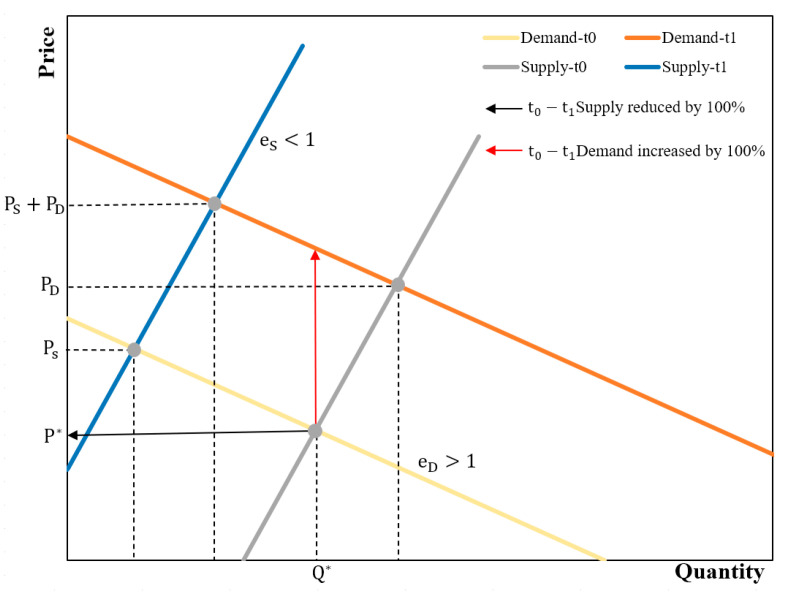
Effects of supply and demand dynamics on the scarcity value of public goods. $P^{*}$ and $Q^{*}$ are the initial equilibrium price and quantity at $t_{0}$ (i.e., 2010); $P_{s}$ is the per-unit ESSV at $t_{0}$ (i.e., 2020) given a change in supply only, holding demand constant. $P_{D}$ is the ESSV given a change in demand only, holding supply constant. $P_{s}+P_{D}$ is the ESSV given a simultaneous change in both supply and demand. $e_{S}$ and $e_{D}$ are the elasticities of supply and demand, respectively.

According to the economic conceptualization, simultaneous changes in supply and demand influence the value of ESs via their effect on relative scarcity. Price elasticity is a key element in changes in the scarcity value. It is the percentage of change in the price in response to quantity changes in supply or demand [64]. More specifically, the directional change in the ES value can be obtained by comparing the relative change in the price with the proportional changes in the quantity supplied and/or quantity demanded. Therefore, we chose the price elasticity of the ESs to measure the price changes as the ESs’ supply and demand change. The price elasticity of demand (supply) for public goods flowing across regions is not large; changes in the quantity demanded of (or supplied by) these ESs are smaller than the proportional change in the price of scarcity, i.e., the elasticity value < 1 [6]. Public goods are non-exclusive and non-rivalrous. Individuals cannot be effectively excluded from their use, and the use by one individual does not reduce the availability to others [65]. Because public goods are indivisible, and each user consumes the same number of goods, additional demand for public ESs will cause a vertical shift in the demand curve of the ESs (Figure 4), increasing the scarcity value. The supply of most public goods can be considered similar to the supply of private goods in the long term; thus, the aggregate supply equals the total supply of all individual suppliers [64,66]. Therefore, an increase in the supply causes a horizontal shift in the public goods supply curve.

The above analysis and previous research on the price elasticity of ESs [67,68,69] show that public ESs have the opposite price elasticities for demand and supply. However, most ESs cannot be traded effectively due to the externalities; thus, it is difficult to obtain their demand price elasticities. Therefore, we used the medium value of the relative change in the scarcity value, as described by Bryan et al. (2018). Two scenarios were designed to calculate the value of ESs: one was affected, and the other was unaffected by changes in supply and demand (Table 4).

#### 3.2.3. Quantifying Changes in Demand for Ecosystem Services

Demand in economics is defined as the desire of consumers who are willing and able to buy at various prices during a given period [70]. It makes sense to measure changes in the demand for ESs using the local population, per capita GDP, and income elasticity [71]. The income elasticity of the demand for ESs is defined as the ratio of the percentage change in demand to the percentage change in income [72]. Previous researchers have calculated the income elasticity of demand for ESs using the equation in Table 5 [6]. We calculated the income price elasticity of various ESs during 2010–2020 using the per capita GDP at the county scale in the YRD.

#### 3.2.4. Scenario Analysis for the Scarcity Value of Ecosystem Services

Based on the analysis of the ESTV and the variables affecting supply and demand, the ESSV is calculated by Equation (8):

(8) $$ESSV_{s,c,t1}=A_{c,t1}\times VC_{s,c}\times \left(1+\Delta p_{s,t1}^{\mathrm{Sup}}+\Delta p_{s,t1}^{\mathrm{Dem}}\right)$$

where $ESSV_{s,c,t1}$ is the scarcity value of ES category s of land use c at time $t_{1}$, $A_{c,t1}$ is the area of land use c at time t, $VC_{s,c}$ is the per-unit value coefficient of ESs in land use c, $\Delta p_{s,t1}^{\mathrm{Sup}}$ is the coefficient of supply-driven relative change in the scarcity value, and $\Delta p_{s,t1}^{Dem}$ is the coefficient of demand-driven relative change in the scarcity value.

## 4. Results

### 4.1. Spatial Distribution Characteristics of the ESTV

**Figure 5:**
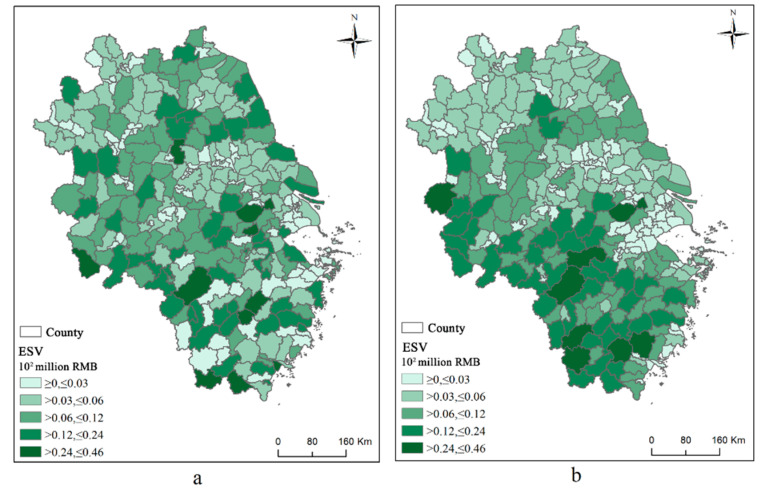
The theoretical value of ecosystem services in the Yangtze River Delta counties in 2010 (**a**) and 2020 (**b**).

Figure 5 shows the spatial characteristics and temporal trends of the ESSV at the county scale in the YRD urban agglomeration from 2010 to 2020. The total ESTV declined from RMB 213 million in 2010 to RMB 196 million in 2020, a decrease of 8.67%. In terms of spatial distribution, counties with ES values of less than 3 million RMB were more dispersed in 2010. There were 94 counties in the central YRD, the Jiangsu, Zhejiang, and Shanghai Ringbelt, and the southwest and southeast regions. The 83 counties with 3–6 million yuan were predominantly located in the northern and eastern coastal cities. Seventy-one counties with 6–12 million yuan were mainly located in the western part of the YRD region, concentrated in Anhui Province. Forty-seven counties with 12–24 million yuan were scattered but were predominantly located in Zhejiang Province. There were only 10 counties with high theoretical supply values in the 24–46 million yuan range. The spatial distribution pattern of the ESSV was different in 2020. High-value areas had shifted to the west and south, and low-value areas had shifted to the central and northern areas and the Jiangsu, Zhejiang, and Shanghai Ringbelt. Specifically, the number of counties within 3 million yuan increased to 112, an increase of 19.15%, with Jiangsu Province being the main contributor to the increase. The number of counties with 12–24 million yuan decreased to 41, a decrease of 12.77%; these were concentrated in the southeast and southern regions. The number of counties with 24–46 million yuan decreased to eight, a decrease of 20%; six counties were in Zhejiang Province, and the other two were in Anhui province and Jiangsu Province, accounting for 75% and 25%, respectively. Although the spatial distribution pattern of the ESSV changed over time, and the location of high-value and low-value areas shifted, the value did not decrease significantly.

**Figure 6:**
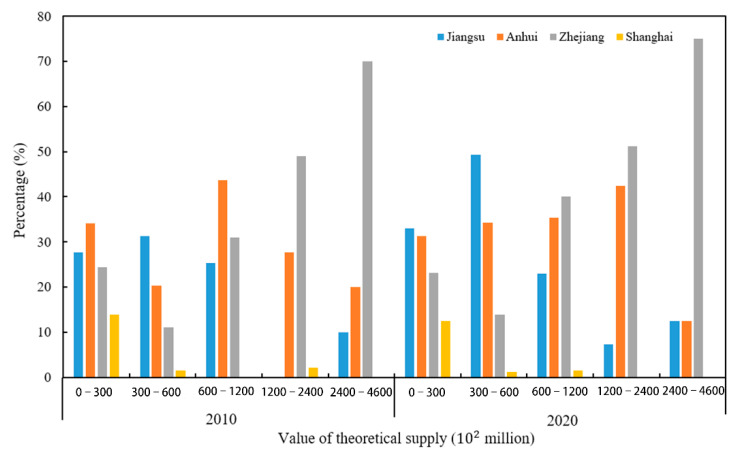
Proportion of counties by province in 2010 and 2020 in different ESTV value ranges.

Figure 6 shows the proportion of the ESTV in each province from 2010 to 2020. Anhui Province had the largest proportion in the 0–3 million RMB range in 2010, whereas Shanghai had the lowest proportion but the highest coverage (13 counties). Jiangsu Province had the biggest proportion in the 3–6 million RMB range, followed by Anhui, Zhejiang, and Shanghai. Anhui Province had the biggest percentage in the 6–12 million yuan range, whereas Zhejiang and Jiangsu had the lowest, but the difference was small. In the 12–24 million RMB range, Zhejiang Province accounted for the highest proportion, followed by Anhui, while Shanghai accounted for a very small part. In the 24–46 million yuan range, Zhejiang Province accounted for the leading share, followed by Anhui and Jiangsu. The ESTV decreased in all provinces from 2010 to 2020, and the proportion of counties in different value intervals changed in each province. In the 0–3 million yuan range, Jiangsu Province overtook Anhui Province and reached first place, and the proportion of counties in Anhui Province, Zhejiang Province, and Shanghai decreased, but the coverage of Shanghai counties was the largest (14). In the 3–6 million RMB range, Jiangsu Province ranked first, followed by Anhui Province, Zhejiang Province, and Shanghai City, and the difference in the proportion of provinces and municipalities increased. In the 6–12 million yuan range, Zhejiang Province was in first place, followed by Anhui, Jiangsu, and Shanghai. In the 12–24 million RMB range, Zhejiang Province and Anhui Province accounted for relatively large proportions, followed by Jiangsu Province. In the 24–46 million RMB range, Zhejiang Province was far ahead, and Anhui and Jiangsu Provinces accounted for an equal share. Overall, the ESTV was low in Shanghai and was more evenly distributed in Anhui, but the peak in Anhui shifted to the higher-value range over time, increasing the contribution to high-value ESs. The contribution to the low-value range increased in Jiangsu Province over time. Forestland, arable land, and water bodies were the land-use categories with high ESTV (more than 12 million RMB), and forestland provided most of the ESs. Despite their small area, water bodies were the primary contributor to high-value ESs because of their high per-unit values of ESs. Arable and forestland comprised the majority of land-use categories with medium ESTVs (6–12 million yuan), and arable land accounted for the largest share. Less than 6 million RMB of ESs was provided by construction land, arable land, and sparsely forested areas.

### 4.2. Spatial Distribution Characteristics of ESSV

**Figure 7:**
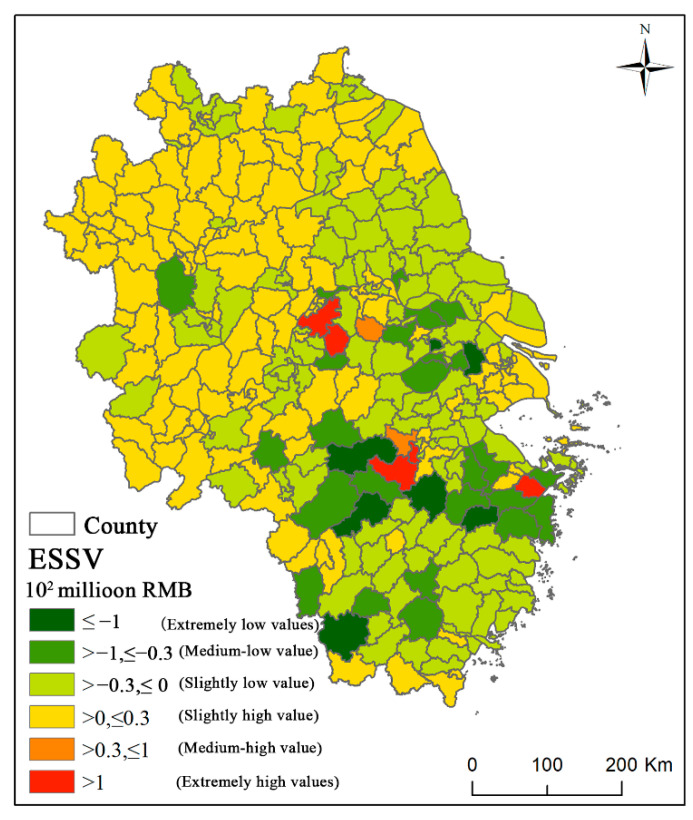
Spatial distribution characteristics of ESSVs based on supply and demand changes in the Yangtze River Delta from 2010 to 2020.

The ESSV increased from 213 million RMB in 2010 to 1.323 billion RMB in 2020, an increase of 521.13%. Figure 7 shows the spatial distribution characteristics of the ESSV based on supply and demand changes in six categories: extremely low value, medium-low value, slightly low value, slightly high value, medium-high value, and extremely high value. The extremely low to medium-low values indicate that the ES supply is much higher than the demand, resulting in a scarcity value much lower than the theoretical supply value. The slightly high to extremely high values indicate that the demand for ES is much higher than the supply, resulting in a scarcity value much higher than the theoretical supply value. First, the spatial distribution characteristics of the ESSV demonstrate that the number of counties in the extremely low and medium-low value categories is small (31), accounting for 2.30% and 7.87% of the total, respectively. These counties are primarily located in the southern region. There are 132 counties in the slightly low-value group, accounting for 43.28% of the total. These counties are along the eastern coast, in the middle and lower sections of the Yangtze River, and in the southern region.

**Figure 8:**
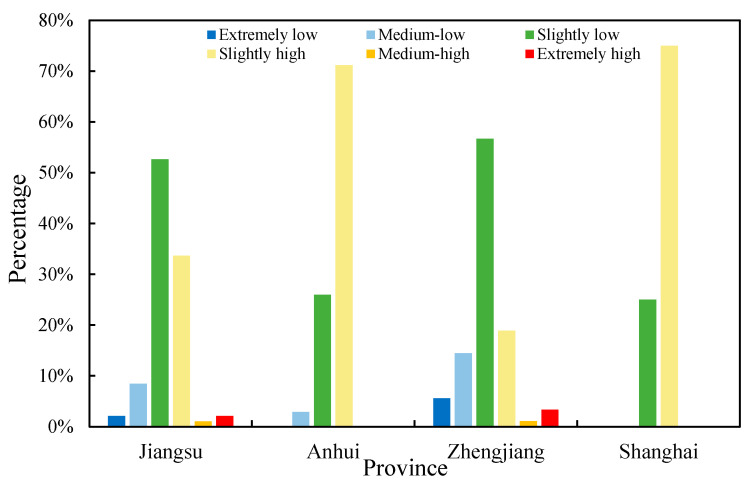
Proportion of ESSVs in six categories in the four provinces in 2020.

Second, the quantitative statistics of the scarcity values in the four provinces shows in Figure 8. The ESSV in Jiangsu province ranges from extremely low to extremely high values, fifty-three percent of the counties have slightly low values, 34% have slightly high values, 8% have medium-low values, 1% have medium-high values, and extremely low- and high-value counties account for 2%, respectively. Seventy-one percent of counties in Anhui Province have slightly high values, 26% have slightly low values, and 3% have medium-low values. Zhejiang Province has values in all categories; 57% of counties have slightly low values, 19% have slightly high values, 14% have medium-low values, 6% have extremely low values, and 3% have extremely high values. The ESSVs in Shanghai fall mostly in the slightly low- and high-value categories, and the proportion of counties with slightly high values is the highest at 75%, while 25% of counties have slightly low values. In general, the difference in the ESSVs is large between counties in Zhejiang Province and Jiangsu Province, and the distribution of ESs is uneven. The difference in the ESSVs between counties in Anhui Province and Shanghai is smaller, and the ESs are more evenly distributed.

Due to high demand and low supply, the ESs are more abundant in areas with high scarcity values, whereas the demand is low and the ESs are not fully utilized in areas with low scarcity values. These two types of ESSVs contrast sharply. The ESSV is closely related to land use, socio-economic development level, and population density. Land-use categories with extremely low and medium-low scarcity values include forest land, arable land, water bodies, and a small amount of construction land; thus, the supply of ESs is adequate. Specifically, two main land-use patterns are observed in the region: one is dominated by construction land with a mix of arable land and water bodies, and the other is dominated by forest land and arable land with a mix of some construction land. Land-use categories with slightly low values include large amounts of forest land, grassland, arable land, water bodies, and construction land. The land-use pattern also includes two types: areas dominated by a network of construction land surrounded by arable land, water bodies, and a small portion of forest land, and the other is dominated by forest land, grassland, and arable land with dispersed construction land. The ESSV in some core urban areas in Jiangsu, Zhejiang, and Shanghai provinces is negative, owing to significant expanses of water bodies and arable land providing many ESs. Some areas have low socioeconomic development levels and low demand, and the supply of ESs outnumbers the demand, lowering the potential value. Land-use categories with slightly high scarcity values include a large amount of arable land, a small amount of forest land, grassland, and construction land. These are mainly located in urban areas with high economic levels or northern areas. They are characterized by a low supply and high demand for ESs. The land-use pattern is dominated by a network of construction land with a mix of arable land, very small amounts of forest land, and water bodies. The other type is dominated by arable land or woodland grassland, with scattered construction land. There is more contiguous construction land, forestland, arable land, and water bodies in the medium-high and extremely high-value regions. They are mainly located in provincial capital city areas. Although the supply of ESs is high, the high level of economic development and population density results in higher ES demand than supply and the high scarcity of these services.

## 5. Discussion

### 5.1. The Effect of Ecosystem Services Scarcity

The decline in the ESTV from 2010 to 2020 indicates a downward trend in the total ESs provided by all land-use categories. The restoration of an ecosystem’s structure and function requires a long time and high costs. It is difficult to restore ecosystems to their former condition when they have surpassed their resilience threshold [25]. Therefore, it is important to respond to a decrease in ESs by taking action to maintain sustainable ecosystems. The high-value areas of the ESTV shifted from north to south, whereas the low-value areas shifted from south to north. This finding is attributed to urbanization and land-use change induced by population migration in different counties. Furthermore, the rising demand for ESs in the context of fast urbanization and economic growth may have resulted in a large increase in the ESSV. People’s willingness to pay for ESs increases as cities and economies develop, but public ESs are dominated by public policy. The price of these ESs is not sensitive to supply and demand changes, leading to small income elasticity of supply, but the scarcity value indicates that the demand has not been met. The demand for ESs far exceeded the supply, particularly in the more economically developed counties of Jiangsu, Zhejiang, and Shanghai provinces, resulting in a much higher ESSV than the ESTV and higher ESSVs than for the other counties. Overall, the large proportion of counties with a slightly high ESSV indicates that the demand for ESs has not been met in the YRD. The proportion of counties with extremely low to slightly low scarcity values accounted for 50%, and there were negative values, indicating that many ESs in the region were not being used efficiently and a significant mismatch between ES supply and demand existed. Our findings are supported by the evaluation and analysis of ES values in the Beijing–Tianjin–Hebei Region based on demand zoning. The total value of ESs decreased at the county scale, and the total and average values decreased from the northern to the southern regions. This spatial variability was related to population density, economic development level, tertiary industry, and education level [47]. From 1990 to 2010, the ESTV in the rapidly urbanizing Guangzhou–Foshan metropolitan area in southern China decreased slightly, while the unmet demand for these services in core urban areas increased. The changes in supply and demand increased the total ESSV by 629%, with woodlands and water bodies providing the highest value of ESs [6]. From 2010 to 2020, the ESTV in the YRD region decreased by 8.6% due to land-use changes, but population growth (3.2%) and per capita GDP growth (134%) led to a 521.13% increase in the scarcity value, representing a significant increase. However, an increase in the scarcity value is undesirable because it indicates a conflict between environmental protection and economic development. Furthermore, the large fluctuations of the scarcity value in the YRD area indicate significant discrepancies between ES supply and demand in urban agglomerations, the low efficiency of ES utilization, and the insufficient exploitation of ecological assets. Differences in the supply and demand of ESs between regions should be coordinated as soon as possible to meet the demand for ESs and economic development in different regions.

### 5.2. Drivers of the Scarcity of Ecosystem Services

The ESSV is affected by changes in the supply and demand for ESs, which are impacted by ecological pressures (exposure areas and intensities) and the structure and function of land use. Specifically, ecological pressures, landscape patterns, location, and area affect ES supply capacity and location, whereas the spatial distribution characteristics of the population, the level of urbanization, and the vulnerability of land-use functions affect the demand for ESs [73]. Urban sprawl has caused many people to work and live in new urban areas, contributing to rapid regional economic development [74,75,76] and expanding residential areas [73]. People require more living space, a variety of lifestyles, different diets, high-quality health care, and multi-level education. Therefore, many natural areas are used for urban development and construction, changing the ecological landscape pattern, location, and area. In addition, human activities interfere with the natural system’s regulation mechanism, increasing ecological pressure and ultimately reducing ES supply [77,78,79,80]. Moreover, population clustering and urbanization increase the demand for ecological services. The key elements of urbanization are population expansion, economic development, and technical progress, which represent and influence the process and patterns of urban development. Population growth increases the demand for ecological services. The fast advancement of modern science and technology will result in significant changes in the human lifestyle and diet, raising the demand for a variety of ESs [81,82]. Increasing wealth and the increasing marginal propensity to consume ESs can strongly influence the price of scarcity, i.e., the higher the income, the higher the likelihood of spending on ESs, especially luxury services [83,84].

### 5.3. Insights for Sustainable Urban Development

The regional system of the human–land relationship theory suggests that natural and human systems interact. As urban development changes the ecosystem, the ecosystem affects urban development. On the one hand, the shrinking quantity and quality of ecosystems lead to a reduction in the supply of ESs, affecting human health and urban safety. For example, more frequent natural disasters, extreme weather, environmental degradation, and urban public safety events pose challenges to healthy and sustainable urban development. Furthermore, unequal ecological and economic growth deepens the gap between urban and rural development, increasing the danger of social instability and disharmony.

The YRD region has launched “Several Policy Measures for Supporting the High-Quality Development of the Yangtze River Delta Ecological Green Integrated Development Demonstration Zone”. This document advocates the transformation of ecological advantages into economic development advantages, promotes the economic revival of the YRD region, and provides policy guarantees for the implementation of reform and innovation in various provinces. The policy requires local governments to take action, such as enhancing the restoration and conservation of ecological land to prevent the loss of urban ecological spaces and ensuring that the material supply of ESs remains sustainable. Stakeholders should take action to optimize production, living, and ecological spaces and improve the environment. For example, stakeholders need to comply with the strict arable land protection system and the red line for ecological protection. Decision-makers may adjust basic farmland planning and perform agricultural land consolidation, construction land consolidation, and rural ecological restoration by implementing comprehensive land improvement platforms and a national coordination mechanism of cross-provincial supplemental arable land. In addition, the government at all levels should actively explore the path of ecological integration, deepen the cooperation model of new-types of urban unify the cross-regional and cross-basin ecological assessment system, and develop cross-regional compensation mechanisms for ecosystems. These measures can be achieved through city function coordination, public service sharing, and collaborative environmental management to improve regional ecological integration and development. Horizontal inter-regional ECP schemes can be formulated regarding the ESSV to reflect the real market demand of ESs and ensure the transformation of ecological resources into ecological capital so that market mechanisms can play a regulatory role. Finally, provincial government decision-makers and related actors should actively pursue the development of a regional green financial service system, effective financial development, and replicable green financial development models through innovations in financial organizations, financing models, service methods, and management systems. Manufacturers should incorporate ecological development goals into the development strategy of enterprises using “industrial ecology and ecological industry” as the development principle. They should refine the ecological performance assessment indices of enterprises. In short, decision-makers should adhere to the principle of people-centered development to meet the urgent needs of the people for a high-quality life.

### 5.4. Limitations

Although this study showed that urban parks and green spaces had a positive effect on the urban environment in terms of ESs, their values were relatively small compared to forests, grasslands, and arable land, and their contribution to the flow of services across regions was relatively small. Urban gardens and urban green spaces were identified as urban construction land in the land-use data. Therefore, the value of ESs provided by urban parks and green spaces was not included. Furthermore, this study focused on the influence of the scarcity value on terrestrial ESs as a result of socio-economic growth and land-use change and did not evaluate marine ESs, which are more complex and for which data are lacking. Future studies should analyze ESs supplied by various land-use categories through data updates and improvement.

## 6. Conclusions

ES value assessments typically only consider changes in the theoretical supply value caused by land-use change. In economics, changes in supply and demand affect the scarcity and scarcity value of a good. The theoretical value of public ESs does not respond to changes in supply and demand in the market, whereas its scarcity value reflects the unmet demand in the market. In addition, scarcity values better reflect the current situation and problems of matching the supply and demand of ESs between regions, providing theoretical guidance for the price tailoring of public ESs across regions. Therefore, it is necessary to assess the ESSV in the context of supply and demand changes. This study established an ESSV model based on changes in supply and demand and applied it to the YRD city cluster in China to investigate the ESSV’s spatial distribution characteristics, identify the supply–demand matching problem, and provide policy recommendations for the YRD’s integrated green development.

Our findings show that the total ESTV decreased by 8.67%, from RMB 213 million in 2010 to RMB 196 million in 2020. The number of counties with an ESTV in the RMB 3–6 million range increased, and that of counties with an ESTV in the other categories decreased. Overall, the ESTV was low in Shanghai and more evenly distributed in Anhui. Jiangsu Province’s contribution to high-value ESs grew over time, whereas Anhui’s contribution to low-value ESs decreased. Although places with high values changed from north to south and those with low values shifted from south to north, there was no significant decrease in the value. These changes were associated with land-use changes caused by urbanization and population migration. The spatial distribution characteristics of the scarcity value showed that rapid urbanization and socio-economic development significantly increased the ESSV. The ESSV increased from 213 million RMB in 2010 to 1.323 billion RMB in 2020, an increase of 521.13%. The scarcity value fluctuated substantially at the county scale, showing a mismatch between the supply and demand of ESs. Counties with extremely low, medium-low, medium-high, and extremely high values accounted for only 12.46% of the total, whereas counties with slightly low and slightly high values accounted for 87.54% of the total. In general, the ESSV difference between counties in Zhejiang Province and Jiangsu Province was high, and the distribution of ESs was uneven. The ESSV difference was low between counties in Anhui Province and counties in Shanghai, and the ESs were more equally distributed. The scarcity value fluctuated substantially at the county scale, showing a mismatch between the supply and demand of ESs. The ESSV was extremely high in counties with high urbanization levels and strong economic dynamics, indicating that ESs were more valuable and important to residents, but they could not obtain adequate ESs. In contrast, the supply of ESs was generally higher and the demand was lower in counties with lower population density and slower economic development, resulting in a negative scarcity value of the ESs and indicating a failure to utilize local ESs.

As a result, it is critical to be aware of the ESSV in metropolitan regions and to adopt appropriate measures to meet the inhabitants’ ecological demands and ensure sustainable urban growth. First, integrating ecosystem planning and management into urban planning and land-use management may reduce ecosystem loss due to rapid urbanization and increase urban resilience and vibrancy. Second, ecosystem planning by regional alliances and management mechanisms should be enhanced, and a cross-regional compensation mechanism for ESs should be developed to encourage the cross-regional flow of ESs and broaden the beneficiary areas. The ESSV should be considered as an ECP standard to highlight differences in the value of ESs under different supply and demand scenarios and to exploit the regulating role of the market mechanisms in regional horizontal EC.

## Figures and Tables

**Table 1:** Ecosystem services that flow across regions.

Types of Ecosystem Services	Spatial Relationships between SPA and SBA
Gas Regulation	Omni-directional
Climate Regulation	Omni-directional
Biodiversity Protection	Omni-directional
Water Containment	Directional

**Table 2:** Locally adjusted per-unit ecosystem service value coefficients for the YRD in 2010. Unit: (yuan/hm^2^).

Land Use Categories		Ecosystem Services				
Primary Categories	Secondary Categories	Gas Regulation	Climate Regulation	Water Containment	Biodiversity Protection	Sum
Arable land	Dry land	11.26	6.05	4.54	2.19	24.04
	Watered land	13.51	9.08	3.63	1.31	27.53
	Paddy field	22.52	12.10	2.27	1.09	37.98
Forestland	Wooded land	78.84	18.36	24.21	10.03	131.44
	Shrubland	63.07	16.52	31.47	8.03	119.09
	Open woodland	47.30	12.85	33.96	6.02	100.13
	Other wooded land	39.42	9.18	36.31	5.02	89.93
Grassland	High cover	18.02	6.12	6.05	3.35	33.54
	Medium cover	14.42	4.90	7.26	2.68	29.26
	Low cover	9.01	3.06	7.87	1.68	21.62
Water bodies	Water bodies	0.00	3.13	154.16	7.66	164.95
Desert	Desert	0.00	0.00	0.23	1.05	1.28
Construction land	Construction land	0.00	0.00	0.00	0.00	0

**Table 3:** Locally adjusted per-unit ecosystem service value coefficients for the YRD in 2020. (yuan/hm^2^).

Land Use Categories		Ecosystem Services				
Primary Categories	Secondary Categories	Gas Regulation	Climate Regulation	Water Containment	Biodiversity Protection	Sum
Arable land	Dry land	9.60	5.16	3.87	1.86	20.49
	Watered land	11.52	7.73	3.09	1.12	23.46
	Paddy field	19.19	10.31	1.93	0.93	32.36
Forestland	Wooded land	67.18	15.64	20.63	8.55	112
	Shrubland	53.75	14.08	26.82	6.84	101.49
	Open woodland	40.31	10.95	28.94	5.13	85.33
	Open woodland	33.59	7.82	30.94	4.28	76.63
Grassland	High cover	15.36	5.21	5.16	2.86	28.59
	Medium cover	12.28	4.17	6.19	2.29	24.93
	Low cover	7.68	2.61	6.70	1.43	18.42
Water bodies	Water bodies	0.00	2.67	131.37	6.53	140.57
Desert	Desert	0.00	0.00	0.19	0.89	1.08
Construction land	Construction land	0.00	0.00	0.00	0.00	0

**Table 4:** Relative changes in the scarcity value under different scenarios.

Scenario Setting	Ecosystem Service Value	Elasticity Factor		Relative Change in Scarcity Value	
		Supply	Demand	Supply $\left(\Delta P_{s,t1}^{Sup}\right)$	Supply $\left(\Delta P_{s,t1}^{Dem}\right)$
Scenario 1	Theoretical value	/	/	0	0
Scenario 2	Scarcity value	0.4	1.6	0.5	0.8

**Table 5:** The income elasticity of demand.

Ecosystem Services	Income Elasticity Functions	Variable Description
Gas Regulation	$y=2\times 10^{-10}x^{2}-4\times 10^{-5}x+1.2649$	x: Per capita GDP of the assessment unit y: Coefficient of income elasticity of demand $\varepsilon_{s,t}$
Climate Regulation	$y=2\times 10^{-10}x^{2}-4\times 10^{-5}x+1.2649$	
Biodiversity Protection	y=0.38	
Water Containment	y=0.45	

## Data Availability

Data sharing is not applicable.
